# Cannabidiol Modulates Neuroinflammatory Markers in a PTSD Model Conducted on Female Rats

**DOI:** 10.3390/biom14111384

**Published:** 2024-10-30

**Authors:** Anna Portugalov, Gaia Peled, Sharon Zorin, Irit Akirav

**Affiliations:** 1 School of Psychological Sciences, Department of Psychology, University of Haifa, Haifa 3498838, Israel; anuta89.ap@gmail.com (A.P.); gaiapeled@gmail.com (G.P.); sharonzorin12@gmail.com (S.Z.); 2 The Integrated Brain and Behavior Research Center (IBBRC), University of Haifa, Haifa 3498838, Israel

**Keywords:** cannabinoids, CBD, inflammation, PTSD, fear extinction

## Abstract

Post-traumatic stress disorder (PTSD) is a debilitating neuropsychiatric condition closely linked to neuroinflammation, with a higher prevalence in women. Cannabidiol (CBD), a non-psychoactive cannabinoid, has shown promise as a potential treatment for PTSD. In this study, we used a PTSD model in which female rats were subjected to a severe foot shock followed by contextual situational reminders (SRs). Testing was conducted one month after exposure. The rats received daily CBD injections for three weeks during the SRs, from days 7 to 28. Two days after the final SR, the rats underwent five extinction trials, followed by the forced swim test (FST). After a five-day rest period, the rats were sacrificed, and brain tissues from the medial prefrontal cortex (mPFC) and ventral subiculum (vSUB) were analyzed for inflammatory markers. Chronic CBD treatment reversed impairments in fear extinction caused by shock and SR. It also reduced learned helplessness in the FST and decreased the upregulation of mPFC-*il1β* induced by shock and SRs. Additionally, exposure to shock and SRs downregulated mPFC-*il6* while upregulating vSUB-*il6.* CBD treatment further downregulated *il6* expression in the vSUB compared to the vehicle groups. Our findings show that CBD effectively inhibited the development of PTSD-like behaviors and suppressed neuroinflammation in the mPFC.

## 1. Introduction

Post-traumatic stress disorder (PTSD) is a complex and chronic neuropsychiatric condition that can develop after exposure to traumatic events or severe stressors. It is strongly associated with inflammation [1,2,3]. In addition to its impact on psychological well-being, PTSD is linked to illness and physical health symptoms [4,5] and cognitive decline [6,7,8]. Multiple studies have shown that individuals diagnosed with PTSD exhibit increased levels of inflammatory markers, including interleukin (IL)-1β, IL-6, and tumor necrosis factor-alpha (TNFα), compared to healthy individuals [1]. These pro-inflammatory cytokines are critical components of the immune system, but when chronically elevated, they contribute to the pathophysiology of PTSD by promoting neuroinflammation [9].

Evidence from pre-clinical studies suggests that exposure to acute or chronic stress may upregulate pro-inflammatory markers in central nervous system regions that regulate the stress response, such as the medial prefrontal cortex (mPFC) and hippocampus. This response highlights sex differences [10]. In humans, both men and women with PTSD exhibit elevated systemic inflammation, though it seems to be more pronounced in women [11]. For example, Ghosh and colleagues [12] found that anxiety-like behavior in both male and female wild-type C57BL/6 mice exposed to a predator odor stress model was associated with increased levels of IL-1β and IL-6 in the hippocampus. Stressed female mice demonstrated a more pronounced anxiety-like phenotype in all behavioral assessments and immune parameters [12]. In another study, only male mice demonstrated upregulated microglial IL-1β and IL-6 genes in the hippocampus following a lipopolysaccharide (LPS) challenge and an inescapable tail shock protocol [13].

Targeting neuroinflammation could be a viable approach for treating PTSD, as research suggests that neuroinflammation significantly contributes to the development of the disorder [14,15,16]. Cannabidiol (CBD), a non-psychoactive cannabinoid known for its anti-inflammatory properties [17], has shown potential as a treatment for PTSD in both pre-clinical and clinical models [18,19,20,21]. CBD acts as an inverse agonist on both cannabinoid receptors (CB1r and CB2r) and inhibits the degrading enzyme fatty acid amide hydrolase (FAAH). This inhibition increases the levels of the endocannabinoid anandamide (AEA) in the synapse [22,23]. Components of the endocannabinoid system (ECS) are present in brain regions linked to stress and fear responses, such as the mPFC and hippocampal areas, where they modulate excitatory and inhibitory signaling in specific neuronal pathways [24]. The therapeutic effects of CBD involve various molecular targets. These include 5-hydroxytryptamine receptor 1A (5HT1A), peroxisome proliferator-activated receptor gamma (PPAR-γ), and a range of receptors such as GABAergic, dopaminergic, cholinergic, and glycinergic receptors. CBD also interacts with transient receptor potential cation channels (TRP channels) and orphan G-protein coupled receptors (GPCRs), including GPR3, GPR6, and GPR55 [25,26,27,28].

In a previous paper [29], we demonstrated that exposure to shock and situational reminders (SRs) leads to significant alterations in emotional processing, evident one week after initial stress exposure. Rats subjected to intense shock combined with SRs exhibited marked avoidance of the shock context, impaired extinction of the traumatic event, and impaired plasticity in the ventral subiculum (vSUB)-nucleus accumbens (Nac) pathway. They also showed increased latency in their startle response. Notably, shock exposure alone did not elicit these effects; rats exposed to shock, regardless of subsequent SR exposure, exhibited avoidance behaviors, heightened anxiety, and hypoalgesia. These findings indicate that SRs significantly exacerbate the impact of shock on both behavioral and physiological outcomes. 

Research from our lab has shown that enhancing endocannabinoid signaling can prevent trauma-like symptoms in a rat model of PTSD involving shock exposure and SRs. Specifically, administering the FAAH inhibitor URB597 following shock exposure and SRs prevented anxiety and depressive-like behaviors in male rats [30,31,32,33]. Additionally, CBD [5 mg/kg, intraperitoneally (i.p.), for three weeks] decreased anxious behavior in the light-dark box test 24 h after foot shock exposure [34]; CBD (10 mg/kg, i.p., administered 30 min prior to behavioral assessment) ameliorated PTSD-like behaviors in C57BL/6 male mice after single-prolonged stress (SPS) and foot shock exposure [35]. Moreover, CBD reversed the SPS-induced downregulation of hippocampal CA1-IL-1β and CA1-TNFα. While most studies emphasize the involvement of the hippocampal CA1 area in stress response and neuroinflammation, the subiculum has been identified as a mediator of the hippocampal–hypothalamo–pituitary–adrenocortical (HPA) axis [36]. Lesion studies suggest that the hippocampus, through the output neurons of the vSUB, plays a role in reducing stress-induced glucocorticoid release [37,38,39].

Most research on trauma and CBD has primarily focused on male subjects. However, it is crucial to explore the effects of CBD on females due to the higher prevalence of PTSD in women [40,41], and the unique hormonal and neuroinflammatory factors that may influence their stress response. Estrogen and progesterone significantly modulate these responses. For example, estrogen is known for its neuroprotective effects [42,43] and can influence HPA axis reactivity during specific menstrual cycle phases [44]. Variability in progesterone levels across the menstrual cycle also affects stress resilience and vulnerability, suggesting that hormonal fluctuations contribute to sex differences in PTSD prevalence [45].

Additionally, female rodents exhibit distinct neuroinflammatory responses to stress, characterized by increased levels of pro-inflammatory cytokines like IL-1β and TNFα [12]. Understanding these differences is crucial for tailoring therapeutic approaches that address sex-specific responses to stress and treatment [46]. There is growing evidence that coping behaviors and physiological responses differ between male and female rodents due to anatomical differences, hormonal influences, and environmental factors [47,48]. For example, Bangasser and Wiersielis (2018) [47] reviewed sex differences in corticotropin-releasing factor (CRF) functions, highlighting female vulnerability to stress-related disorders. Moreover, estrogen’s impact on endocannabinoid levels and receptor expression contributes to observed sex differences in cannabinoid effects on the brain [49,50].

In this study, we utilized a PTSD model in which rats were subjected to a severe foot shock in an inhibitory avoidance apparatus, followed by contextual SRs of the shock, with testing conducted a month post-exposure [30,31,51]. We hypothesized that chronic administration of CBD for three weeks would prevent PTSD-like symptoms and reduce the activation of neuroinflammatory markers in the mPFC and vSUB in female rats exposed to the shock and reminders model of PTSD.

## 2. Materials and Methods

### 2.1. Subjects

Thirty-six female Sprague Dawley (SD; Envigo, Jerusalem, Israel) rats were housed in polypropylene cages (59 × 28 × 20 cm), with 4–5 animals per cage. The rats were maintained at 22 ± 2 °C under a 12 h light/dark cycle (lights on at 7 a.m.). The sample size was determined based on our previous study, which included males and females in a PTSD model [52]. The rats had ad libitum access to water and laboratory rodent chow. The experiment received approval from the University of Haifa Ethics and Animal Care Committee (approval number: UoH-IL-2201-106–4). We implemented all necessary measures to minimize pain or discomfort while adhering to the 3Rs principles. Replacement: we aimed to examine complex behaviors such as fear extinction and learned helplessness in response to stress. These processes cannot be effectively studied in invertebrates. Reduction: the sample size was based on previous work [52], using the smallest number of animals per group necessary to obtain reliable results. The experiment was performed without replicates. Refinement: the rats underwent a three-day acclimation period after their arrival to help them adapt to their new environment. An enriched housing environment was provided. All injections, behavioral tests, and decapitations were performed by experienced experimenters.

### 2.2. Shock and Situational Reminders (SRs)

The stress paradigm is based on our previous studies [30,33,53]. Rats were exposed to the stressor in a passive avoidance apparatus (50 × 25 × 30 cm; constructed by the University of Haifa workshop), which was divided into two equal-size compartments separated by an automatic guillotine door.

On the shock exposure day, rats were placed in the light compartment. After two minutes of exploration, the guillotine door opened, allowing access to the dark compartment. Thirty seconds after entering, the door closed, and the rat received a 1.5 mA shock for ten seconds. Following the shock, the rats remained in the dark side for an additional twenty seconds before being returned to their home cages. The no-shock groups received the same treatment, with the shock mechanism inactivated.

For SRs, rats spent one minute in the light start chamber with the gate closed to prevent entry into the shock compartment, avoiding extinction. SRs occurred four times every seven days over 28 days, specifically on days 7, 14, 21, and 28.

For extinction (Ext), rats were put back in the light compartment until they crossed over to the dark side of the shuttle box, undergoing five days of extinction training. If a rat did not cross within 300 s, the experimenter gently guided it to the dark side. The opening between the two sides was then blocked and no foot shock was administered. The rat freely explored the dark side for 180 s before returning to their home cage. We measured the latency to cross over to the dark side in seconds.

### 2.3. Drug Treatment

Rats received daily i.p. injections of either vehicle (1 mL/kg) or CBD (10 mg/kg) during the SR period, from day 7 to 28, at 9:00 a.m. Control rats were injected with the vehicle only. The drugs were dissolved in a solution of 2% Tween-80 and 98% saline (0.9% NaCl). The dosage was based on previous research from our lab [52,54].

### 2.4. Estrous Cycle

On the shock day and the first day of extinction, we measured the estrous cycle. Vaginal cytology samples were collected by gently introducing and extracting a small amount of phosphate buffer from the rat’s vagina using a micropipette. The cycle stage (metaestrous, diestrous, pro-estrous, or estrous) was determined by examining the presence of leukocytes, nucleated epithelial cells, or cornfield epithelial cells [52].

### 2.5. Forced Swim Test (FST)

To assess learned helplessness as a depressive-like behavior, we performed the FST. The test was conducted in a cylindrical water tank (62 cm in diameter, 40 cm in height) filled with water at 22 °C. The tank was illuminated with red light, and the water level was adjusted to prevent the rats from reaching the bottom with their hind paws. Rats were exposed to the swim tank for 15 min on the first day and 5 min on the second day. Videos from the second day of each FST session were analyzed for passive coping (immobility) and active coping (climbing and swimming) behaviors [55].

### 2.6. Real-Time (RT) PCR

Rats were decapitated, and brain tissues from the mPFC and vSUB were collected for molecular analysis (see Supplementary Materials; Figure S1). RNA extraction, cDNA synthesis, and qRT-PCR were performed following methods previously detailed [56,57]. Briefly, 1000 ng of RNA was reverse-transcribed into cDNA using the qScript cDNA Synthesis Kit (Quanta Biosciences, Gaithersburg, MD, USA). Subsequently, qRT-PCR was then conducted using RT SYBR Green for amplification, following the manufacturer’s protocol, with specific primers from Quanta Biosciences (Quanta Biosciences, Gaithersburg, MD, USA). Reactions were run on a Step One RT-PCR system (Applied Biosystems, Waltham, MA, USA). Fold-changes in gene expression were calculated using the ddCt method, normalized to the housekeeping gene hypoxanthine phosphoribosyl transferase (HPRT). Primers (see Table 1) were designed and synthesized by Agentek (Tel Aviv, Israel), and their suitability was verified through standard curve analysis, melting curve analysis, and evaluations of linearity and threshold [58].

### 2.7. Experimental Design

**Scheme 1:**
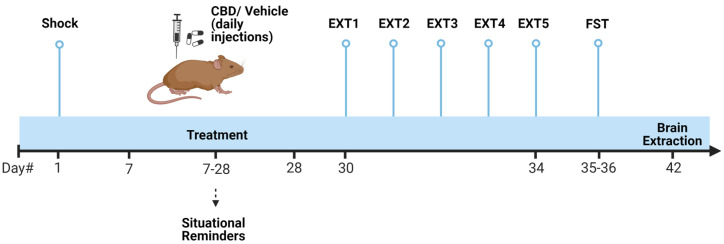
Experimental design. Female rats were subjected to a severe foot shock (1.5 mA, 10 s) followed by exposure to four one-minute contextual situational reminders (SRs) on days 7, 14, 21, and 28. CBD (10 mg/kg) or vehicle (1 mL/kg) were administered i.p. for three weeks. From days 30 to 34, rats underwent 5 extinction (Ext) trials, followed by the forced swim test (FST). On day 42, the rats were sacrificed, and brain tissues from the mPFC and vSUB were collected for mRNA analysis of inflammatory genes (*il1β*, *il6*, and *tnfα*).

On day 1 (see Scheme 1), female rats received a single foot shock (1.5 mA, ten seconds) in an inhibitory avoidance apparatus. They were then exposed to four contextual one-minute SRs on days 7, 14, 21, and 28. CBD (10 mg/kg) or vehicle (1 mL/kg) were administered i.p. for three weeks from day 7 to 28. On day 30, rats underwent five trials of Ext, with 24 h intervals between each trial, followed by the FST. After five days of rest, the rats were sacrificed, and brain tissues from the mPFC and vSUB were extracted for mRNA analysis.

### 2.8. Statistical Analysis

The results are expressed as means ± SEM. For statistical analysis, one-way ANOVA, two-way ANOVA, repeated measures ANOVA, and Pearson bivariate correlation tests were employed as indicated. All post hoc comparisons were conducted using independent-samples *t*-tests. Significance was set at *p* ≤ 0.05. Data were analyzed using SPSS version 27 (IBM, Chicago, IL, USA). The assumption of normality was assessed using the Kolmogorov–Smirnov and Shapiro–Wilk tests.

## 3. Results

### 3.1. The Effect of CBD on Behavior in Female Rats Exposed to Shock and SRs

**Figure 1:**
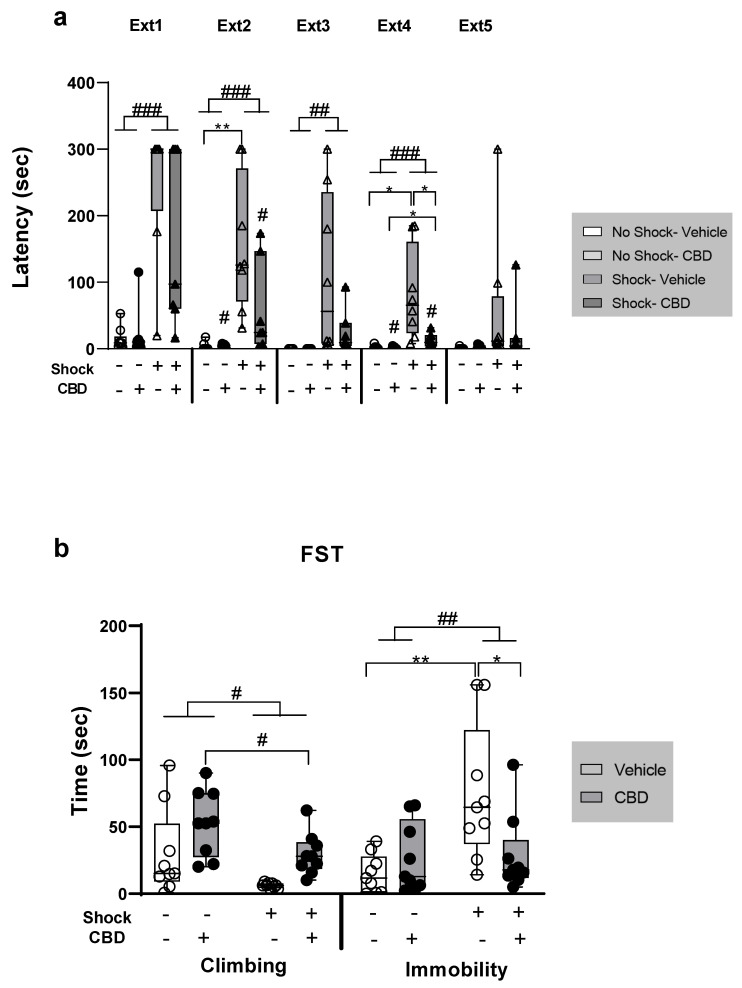
The effect of CBD on behavior in female rats exposed to shock and situational reminders (SRs). (**a**) On day 30, rats underwent five extinction (Ext) trials, with 24 h intervals between each trial. During each trial, rats were allowed to explore the dark chamber for 180 s before being returned to their home cages. Latency to cross into the dark chamber (in seconds) was measured. Shocked groups exhibited increased latency to enter the dark chamber compared to no-shock groups across extinction (Ext) days 1–4. On Ext days 2 and 4, the shock–vehicle group showed a significantly higher latency compared to the no-shock–vehicle group. On Ext day 4, the shock–vehicle group also exhibited increased latency compared to the shock–CBD group. Group sizes: No-shock–vehicle and no-shock–CBD: n = 9; shock–vehicle: n = 8; shock–CBD: n = 7. (**b**) On days 35–36, a forced swim test (FST) was conducted. Rats were exposed to the swim tank for 15 min on the first day and 5 min on the second day. Videos from the second day were analyzed to assess active coping (climbing) and passive coping (immobility). For climbing behavior (**left**), non-stressed and CBD-treated females exhibited more climbing (i.e., active coping). Exposure to shock and SRs increased immobility compared to no-shock groups (**right**); the shock–vehicle group exhibited significantly higher immobility compared to both the shock–CBD and no-shock–vehicle groups. Group size: n = 9 for each group. *, *p * < 0.05; **, *p * < 0.01 indicate statistically significant effects followed by post hoc comparisons; #, *p* < 0.05; ##, *p * < 0.01; ###, *p * < 0.001 indicate statistical significance in main effects.

For Ext (Figure 1a), a repeated measures ANOVA (shock × drug × Ext day; 2 × 2 × 5) revealed significant effects of drug (F_(1,116)_ = 10.38, *p* = 0.003), shock (F_(1,116)_ = 52.66, *p* < 0.001), Ext (F_(4,116)_ = 14.26, *p* < 0.001), shock × drug (F_(1,116)_ = 11.27, *p* = 0.002), and shock × Ext (F_(4,116)_ = 9.45, *p* < 0.001) interactions. Post hoc analysis showed that the shock groups exhibited increased latency to enter the dark chamber compared to the no-shock groups on Ext 1 and 2 (*p* < 0.001), Ext 3 (*p* = 0.005), and Ext 4 (*p* = 0.003), but not on Ext 5. These results suggest that exposure to shock and SRs impaired extinction. An independent samples *t*-test found that the shock–vehicle group showed increased latency compared to the no-shock–vehicle group on Ext 2 (t_(7.045)_ = −4.269, *p * = 0.004) and Ext 4 (t_(7.015)_ = −2.763, *p* = 0.028). Additionally, on Ext 4, the shock–vehicle group exhibited increased latency compared to the shock–CBD group (t_(7.327)_ = 2.331, *p =* 0.051), suggesting that CBD may facilitate extinction in rats exposed to shock and SRs. A difference was also found between the shock–CBD group and the no-shock–CBD group, with the shock–CBD group exhibiting higher latency times (t_(6.234)_ = −2.795, *p* = 0.03).

For freezing behavior during SRs see Figure S2 in the Supplementary Materials.

For climbing in the FST (Figure 1b, left), a two-way ANOVA [shock × drug (2 × 2)] revealed significant effects of both shock (F_(1,32)_ = 7.14, *p* = 0.012) and drug (F_(1,32)_ = 7.14, *p* = 0.012). However, there was no significant shock × drug interaction (F_(1,32)_ = 0.005, *p* = 0.943). The groups that received no shock and those treated with CBD exhibited increased climbing, indicating more active coping behavior.

For immobility in the FST (Figure 1b, right), a two-way ANOVA revealed a significant effect of shock (F_(1,32)_ = 7.78, *p* = 0.009) and a significant shock × drug interaction (F_(1,32)_ = 6.75, *p* = 0.014), but no significant effect of drug alone (F_(1,32)_ = 2.57, *p* = 0.118). The shock group exhibited increased immobility (i.e., passive coping) compared to the no-shock group. An independent samples *t*-test showed that the shock–vehicle group demonstrated increased immobility compared to both the shock–CBD group (t_(16)_ = 2.32, *p* = 0.034) and the no-shock–vehicle group (t_(9.025)_ = −3.236, *p* = 0.01). This suggests that exposure to shock and SRs increased passive coping, while CBD treatment normalized this behavior.

### 3.2. Effects of CBD on mRNA Expression of Inflammatory Genes in Rats Exposed to Shock and SRs in the mPFC and vSUB

Following the behavioral tests, we examined the expression of the inflammatory genes *il1b*, *il6*, and *tnfa* in the mPFC and vSUB. All analyses were conducted using a two-way ANOVA [shock × drug (2 × 2)].

#### 3.2.1. *Il1β*

**Figure 2:**
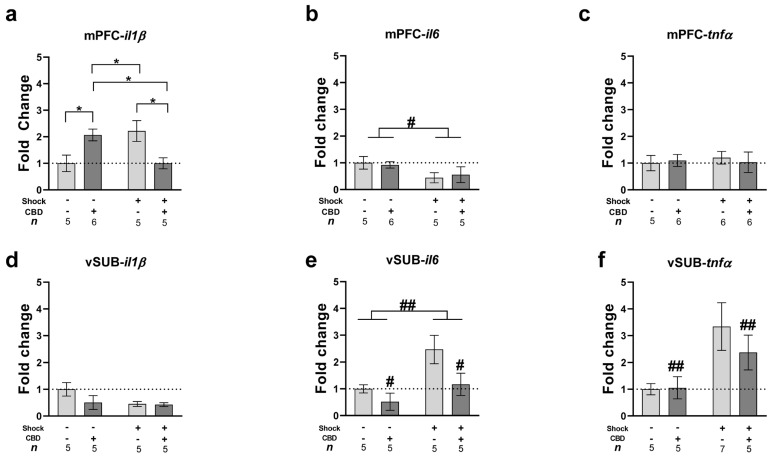
Effects of CBD on mRNA expression of inflammatory genes (*il1β*, *il6*, and *tnfα*) in rats exposed to shock and SRs in the mPFC and vSUB. Rats were sacrificed on day 42, following five days of rest after the FST. (**a**) Chronic CBD treatment normalized the upregulation of mPFC-*il1β* induced by shock and SRs. Additionally, CBD increased mPFC-*il1β* expression in non-stressed groups compared to vehicle. (**b**) Exposure to shock and SRs downregulated mPFC-*il6* expression, with no effect of CBD treatment. (**c**) No significant differences were found in mPFC- *tnfα* expression. (**d**) Similarly, no significant differences were found in vSUB-*il1β* expression. (**e**) Exposure to shock and SRs upregulated vSUB-*il6* expression compared to non-stressed females, an effect attenuated by CBD treatment. (**f**) CBD treatment downregulated vSUB-*tnfα* expression in stressed females compared to the vehicle group. *, *p * < 0.05 indicates statistically significant effects followed by post hoc comparisons; #, *p* < 0.05; ##, *p * < 0.01 indicate statistical significance in main effects.

In the mPFC (Figure 2a), a two-way ANOVA revealed a significant shock × drug interaction effect (F_(1,17)_ = 13.2, *p* = 0.002), with no significant main effects of shock (F_(1,17)_ = 0.04, *p* = 0.832) or drug (F_(1,17)_ = 0.3, *p* = 0.591). An independent samples *t*-test showed an upregulation of mPFC-*il1β* in the shock–vehicle group compared to both the no-shock–vehicle (t_(8)_ = 2.373, *p* = 0.045) and shock–CBD (t_(8)_ = −2.73, *p* = 0.026) groups, suggesting that CBD normalized the shock- and SRs-induced mPFC-*il1β* upregulation. Additionally, the no-shock–CBD group exhibited increased mPFC-*il1β* expression compared to both the no-shock–vehicle (t_(9)_ = 2.366, *p* = 0.042) and shock–CBD (t_(9)_ = −2.776, *p* = 0.022) groups. This indicates a differential effect of CBD in stressed and non-stressed females.

In the vSUB (Figure 2d), a two-way ANOVA did not reveal any significant effects of shock (F_(1,16)_ = 1.75, *p* = 0.205), drug (F_(1,16)_ = 0.838, *p* = 0.374), or the shock × drug interaction (F_(1,16)_ = 2.19, *p* = 0.158).

#### 3.2.2. *Il6*

In the mPFC (Figure 2b), a two-way ANOVA revealed a significant effect of shock (F_(1,17)_ = 5.45, *p* = 0.032), with no significant effects of the drug (F_(1,17)_ = 0.067, *p* = 0.799) or shock × drug interaction (F_(1,17)_ = 0.31, *p* = 0.586). Exposure to shock and SRs downregulated the expression of mPFC-*il6* compared to non-stressed females.

In the vSUB (Figure 2e), a two-way ANOVA revealed significant effects of shock (F_(1,16)_ = 9.46, *p* = 0.007) and drug (F_(1,16)_ = 6.33, *p* = 0.023), with no significant effect of the shock × drug interaction (F_(1,16)_ = 0.02, *p* = 0.873). Exposure to shock and SRs upregulated vSUB-*il6* expression compared to non-stressed females. Additionally, CBD downregulated vSUB-*il6* expression compared to the Vehicle groups.

#### 3.2.3. *Tnfα*

In the mPFC (Figure 2c), a two-way ANOVA did not reveal any significant effects of shock (F_(1,18)_ = 0.06, *p* = 0.801), drug (F_(1,18)_ = 0.01, *p* = 0.901), or the shock × drug interaction (F_(1,18)_ = 0.27, *p* = 0.609).

In the vSUB (Figure 2f), a two-way ANOVA revealed a significant effect of the drug (F_(1,20)_ = 8.66, *p* = 0.008), with no significant effects of shock (F_(1,20)_ = 2.54, *p* = 0.127) or the shock × drug interaction (F_(1,20)_ = 2.54, *p* = 0.127).

### 3.3. Correlations Between the Expression of Inflammatory Genes and Behavior

Pearson bivariate correlations were conducted to explore associations between the expression of inflammatory genes (*il1β*, *il6* and *tnfα*) and behavior (Ext average and climbing and immobility in the FST) (see Table 2). An average was calculated for all five days of Ext.

In the mPFC, negative correlations were found between *il6* expression and Ext average (r = −0.728, *p* < 0.001) and immobility in the FST (r = −0.61, *p* = 0.004). This suggests that decreased mPFC-*il6* is associated with increased latency to cross over to the dark chamber (i.e., impaired extinction behavior) and increased learned helplessness (i.e., depressive-like behavior), respectively.

In the vSUB, positive correlations were found between *il6* expression and Ext average (r = 0.497, *p* = 0.042) and immobility in the FST (r = 0.462, *p* = 0.04). This suggests that increased vSUB-*il6* is associated with impaired extinction and increased learned helplessness, respectively. Additionally, a positive correlation was found between *tnfα* expression and Ext average (r = 0.483, *p* = 0.031), and a negative correlation between *tnfα* expression and climbing behavior in the FST (r = −0.666, *p* = 0.001). This suggests that increased vSUB-*tnfα* is associated with impaired extinction and decreased climbing behavior (i.e., less active coping), respectively.

The distribution of estrus phases in each group of female rats was observed on the shock day and the first day of extinction. A similar distribution of rats across the diestrus, proestrus, estrus, and metestrus phases was noted within each group, with no significant correlation found between estrus phase and the latency to enter the dark chamber on extinction day 1. For detailed estrus phase distribution and correlation data, refer to Table S1 and Table S2, respectively.

## 4. Discussion

In this study, we investigated the therapeutic effects of CBD on anxious- and depressive-like behaviors in female rats exposed to shock and SRs. We demonstrated that chronic CBD treatment reversed impairments in fear extinction and decreased learned helplessness in the FST caused by shock and SRs. Additionally, shock and SR exposure upregulated *il1β* and downregulated *il6* in the mPFC, while upregulating vSUB-*il6.* CBD treatment did not affect *il6* in the mPFC but downregulated *il6* in the vSUB compared to vehicle groups. The shock and SR-induced reduction in mPFC-*il6* and the increase in vSUB-*il6* were associated with impaired extinction behavior and increased learned helplessness. Increased *tnfα* in the vSUB was also associated with impaired extinction and decreased active coping. These findings align with the notion that neuroinflammation is associated with anxiety and depression [59,60]. No significant correlation was found between mPFC-*il1β* upregulation and anxious- or depressive-like phenotypes. Our results suggest that CBD’s therapeutic effects may be linked to its ability to modulate specific neuroinflammatory pathways, particularly by regulating the expression of key cytokines in brain regions associated with emotional regulation and stress responses. The observed changes in *il6* and *tnfα* in the vSUB, along with their correlation with behavior, suggest that CBD may exert its effects by targeting neuroinflammatory processes that contribute to the development and persistence of PTSD-like symptoms.

Women are twice as likely as men to develop PTSD, with female sex recognized as a significant risk factor for PTSD following psychological trauma [40,41]. Hormonal differences between men and women influence the activation of the HPA axis and the release of pro-inflammatory cytokines following stress exposure [61]. For instance, higher levels of progesterone have been linked to an increased ability to recall negative memories [62], while circulating levels of 17-β estradiol are associated with impaired fear extinction [63]. Despite this, a significant gap remains in research on sex-specific neuroimmune changes. Our study can shed light on the neuroinflammatory responses to stress, specifically in females, using a PTSD model previously applied to males. This study may enhance our understanding of sex differences in the neuroinflammatory response to the same stressor (i.e., foot shock and SRs).

Pre-clinical and clinical studies indicate that various types of stress can impair extinction [64,65]. Showing that CBD restored fear extinction in rats exposed to shock and SRs is consistent with previous studies in different animal models of stress [66,67,68,69]. Additionally, our results indicate that CBD treatment reversed the decrease in climbing behavior and the increase in immobility in the FST, aligning with previous findings [70,71,72] and data from our lab [73]. This suggests that CBD may also exert antidepressant-like effects, as chronic CBD treatment (10 mg/kg, i.p.) has been shown to reverse the increased immobility in the FST induced by unpredictable chronic mild stress (UCMS) in male rats [73].

Many animal models of PTSD focus on immune changes found in the mPFC and hippocampal areas [11]. IL-1β and IL-6 are pro-inflammatory markers that play crucial roles in promoting inflammation and facilitating the recruitment and activation of immune cells at infection sites [74]. However, elevated levels of these cytokines can lead to neuronal damage and cell death [75,76]. Wang et al. (2018) [77] reported an upregulation of mPFC-*il1β* mRNA in male rats exposed to the SPS model of PTSD. Similarly, another study found that IL-1β and IL-6 were upregulated in the mPFC and hippocampus 42 days after male mice were subjected to a resident aggressor model for 5 or 10 consecutive days, although the specific hippocampal areas examined were not detailed [78]. Interestingly, our findings in the mPFC, where *il1β* was upregulated and *il6* downregulated, contrast with previous results from our lab using the same model in male rats [79]. In that study, we observed downregulation of mPFC-*il1β* and upregulation of mPFC-*il6*. This discrepancy suggests that the model may affect stress reactivity and neuroinflammation in a sex-dependent manner, supporting other studies that highlight the importance of stressor context and sex in vulnerability to PTSD-like symptoms [80].

Experimental evidence suggests differential expression of microglia in the PFC and subiculum following stress exposure [81,82,83]. For instance, microglial activation in the PFC has been associated with the regulation of synaptic plasticity, which is critical for learning and memory processes, including fear extinction [84]. Conversely, the subiculum is involved in integrating contextual information and modulating emotional responses [85,86], and stress-induced changes in microglial activity in this area may affect the encoding of extinction memory.

Importantly, our study demonstrates that chronic CBD treatment normalized the upregulation of mPFC-*il1β* in stressed females. This finding aligns with other animal models of neuropsychiatric disorders, which have shown the neuroprotective and anxiolytic effects of CBD [25,28,35,87]. Most of these studies were performed on males, suggesting that there may not be significant sex differences in the neuropsychiatric effects of CBD. According to a recent review [26], the only consistent sex difference reported in multiple studies is the potential antidepressant-like effects of CBD in male rodents, not in females.

Another notable finding is that exposure to shock and SRs induced upregulation in vSUB-*il6,* and CBD treatment decreased its levels in both stressed and non-stressed females. Herman and Mueller (2006) [36] demonstrated the importance of the vSUB in stress responsiveness by inhibiting the HPA axis. However, literature on neuroinflammation in the vSUB due to stress exposure is limited. Our study shows that stress affects *il6* expression and that these changes are associated with behavioral phenotypes. Specifically, increased vSUB-*il6* was associated with impaired extinction and increased depressive-like behavior, suggesting that CBD may ameliorate PTSD-like behaviors by modulating vSUB-*il6*.

Studies have shown that isolated CBD offers several important benefits, including the absence of psychoactive or anxiety-inducing effects typically associated with endocannabinoid system activation, a lack of tolerance or dependence development, and its safety at elevated doses in both humans and animals [88]. Research involving human subjects has highlighted the potential therapeutic effects of CBD in treating PTSD [18]. For example, patients who took daily oral CBD for 8 weeks showed a reduction in PTSD symptom severity, reflected by a consistent decrease in their mean PTSD checklist for DSM-5 scores [19]. Additionally, CBD was found to decrease the frequency of nightmares in patients experiencing this symptom. A case study from 2016 also reported relief in PTSD symptoms, including reduced anxiety and improved sleep, in a ten-year-old child with PTSD [89]. However, it is important to examine the impact of CBD not only on behavior but also on underlying brain mechanisms, such as hormonal changes and neuroinflammation in specific brain areas. This knowledge can be gained through the use of animal models.

We also found that chronic CBD treatment altered the expression of neuroinflammatory markers in non-stressed female rats, upregulating mPFC-*il1β* and downregulating vSUB-*il6*, suggesting that CBD may also affect the non-stressed brain.

In summary, our study highlights the sex differences in the neuroinflammatory response to shock and reminders model. A related study from our lab [79] explored the effects of MDMA treatment in male rats using the same PTSD model. The male rats exhibited similar behavioral patterns to those observed in the current study, including impaired extinction [79]. However, distinct neuroinflammatory patterns emerged: males exhibited downregulated mPFC-il1β and upregulated mPFC-il6, whereas females showed the opposite pattern. Notably, CBD reversed the mPFC-il1β upregulation only in females. Although prior studies [26] suggested that CBDs antidepressant-like effects are limited to males, our findings reveal its potential to reverse learned helplessness in females as well, highlighting the need for sex-specific approaches in PTSD treatment.

## 5. Conclusions

Our shock and reminders model demonstrated sustained effects on behavior and neuroinflammation, persisting for over a month after the initial shock. This suggests a chronic neuroinflammatory phenotype that more closely resembles what is observed in human PTSD patients [14,90]. Notably, CBD inhibited the development of PTSD-like behaviors in our rat model and provided long-term suppression of neuroinflammation, underscoring its potential value in clinical applications.

## Figures and Tables

**Table 1:** Primers for mRNAs used in RT-PCR. On day 42, rats were sacrificed, and brain tissues from the mPFC and vSUB were collected for the analysis of inflammatory gene expression, specifically il1β, il6, and tnfα. The housekeeping gene hprt was used as a reference gene. F: forward primer; R: reverse primer.

Name	Description	Gene Bank ID (NM)	Protein Name	Primer Sequence
*hprt*	Housekeeping gene; used as a reference gene	NM_012583.2	HPRT	F: 5′GAGCACTTCAGGGATTTGAATCA3′ R: 5′GTAGATTCAACTTGCCGCTGCTGTCT3′
*Il1β*	Interleukin 1 beta	NM_031512.2	IL-1beta	F: 5′GCTGTGGCAGCTACCTATGTCTT3′ R: 5′GTCACAGAGGACGGGCTCTTC3′
*Il6*	Interleukin 6	NM_012589.2	IL-6	F: 5′CTTCCAAACTGGATATAACCAGG3′ R: 5′CTTCACAAACTCCAGGTAGAAAC3′
*tnfα*	Tumor necrosis factor alpha	NM_012675.3	TNF-alpha	F: 5′CCAGACCCTCACACTCAGATC3′ R: 5′CTCCGCTTGGTGGTTTGCTA3′

**Table 2:** Correlations between the expression of inflammatory gene and behavior. Correlational analyses were performed to explore the link between the levels of inflammatory genes (il1β, il6, and tnfα) and behavior. This included the average time it took for extinction (Ext average) and behaviors like climbing and immobility during the forced swim test (FST). These analyses aimed to explore the potential associations between anxious- and depressive-like phenotypes and the expression of inflammatory genes in both the mPFC and vSUB. The Ext average was calculated across all five extinction trials. Ext: extinction; FST: forced swim test; mPFC: medial prefrontal cortex; vSUB: ventral subiculum.

	Ext. Average	FST-Climbing	FST-Immobility
mPFC-*il1β*	r = 0.113 *p* = 0.625	r = −0.152 *p* = 0.51	r = 0.238 *p* = 0.3
mPFC-*il6*	r = −0.728 *p * < 0.001	r = 0.281 *p* = 0.231	r = −0.61 *p* = 0.004
mPFC-*tnfα*	r = −0.045 *p* = 0.845	r = −0.113 *p* = 0.617	r = −0.018 *p* = 0.938
vSUB-*il1β*	r = −0.237 *p* = 0.344	r = −0.313 *p* = 0.179	r = −0.273 *p* = 0.244
vSUB-*il6*	r = 0.497 *p* = 0.042	r = −0.414 *p* = 0.069	r = 0.462 *p* = 0.04
vSUB-tnfα	r = 0.483 *p* = 0.031	r = −0.666 *p* = 0.001	r = 0.391 *p* = 0.072

## Data Availability

Data are contained within the article and Supplementary Materials.

## Supplementary Materials

The following supporting information can be downloaded at: https://www.mdpi.com/article/10.3390/biom14111384/s1, Figure S1: A coronal view atlas illustration of brain areas extracted for mRNA analysis; Figure S2: Freezing behavior during the situational reminders (SRs); Table S1: Distribution of estrus phases in female rats on shock day and the first day of extinction; Table S2: Pearson bivariate correlation between estrus phase on the first day of extinction and the latency to enter the dark chamber on Ext 1 in shock- and situational reminders-exposed female rats.
