# Supplementary material for: Cannabidiol Modulates Neuroinflammatory Markers in a PTSD Model Conducted on Female Rats
Source: Biomolecules. 2024 Oct 30;14(11):1384. doi: 10.3390/biom14111384 (PMC11591736; doi:10.3390/biom14111384)
Supplement: Supplementary file 1 [file biomolecules-14-01384-s001.zip › biomolecules-3198081-supplementary.pdf]

Supplementary information

Material and Methods

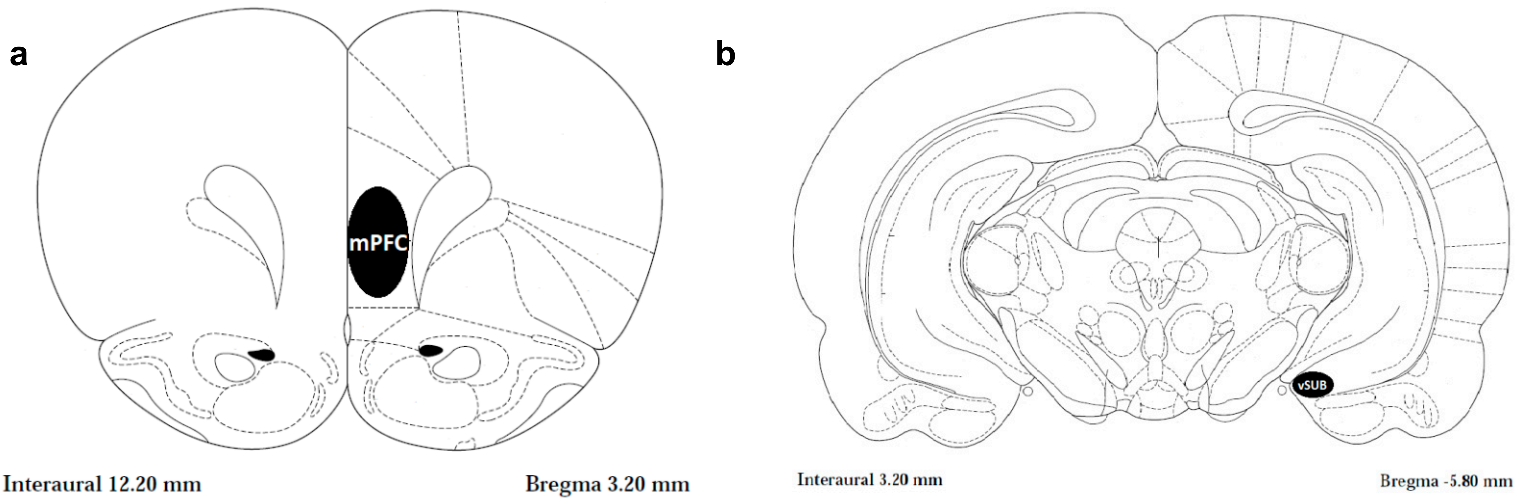

Figure S1. A coronal view atlas illustration of brain areas extracted for mRNA analysis. Samples were obtained using 1 mm diameter punches. The numbers indicate the distance from Bregma (Paxinos & Watson, 2007): (a) the mPFC at 3.20 mm anterior to bregma. (b) the vSUB at -5.80 mm posterior to bregma. mPFC: medial prefrontal cortex; vSUB: ventral subiculum.

Results

For situational reminders (SRs; Figure S2), a repeated measures ANOVA (shock × drug × SR; 2 × 2 × 4) revealed significant effects of shock ($F_{(1,87)} = 14.47$, $p < 0.001$) and shock × SR interaction ($F_{(3,87)} = 12.59$, $p < 0.001$). Post hoc analysis showed that the shock groups exhibited increased freezing behavior compared to the no-shock groups ($F_{(1,29)} = 11.5$, $p = 0.002$).

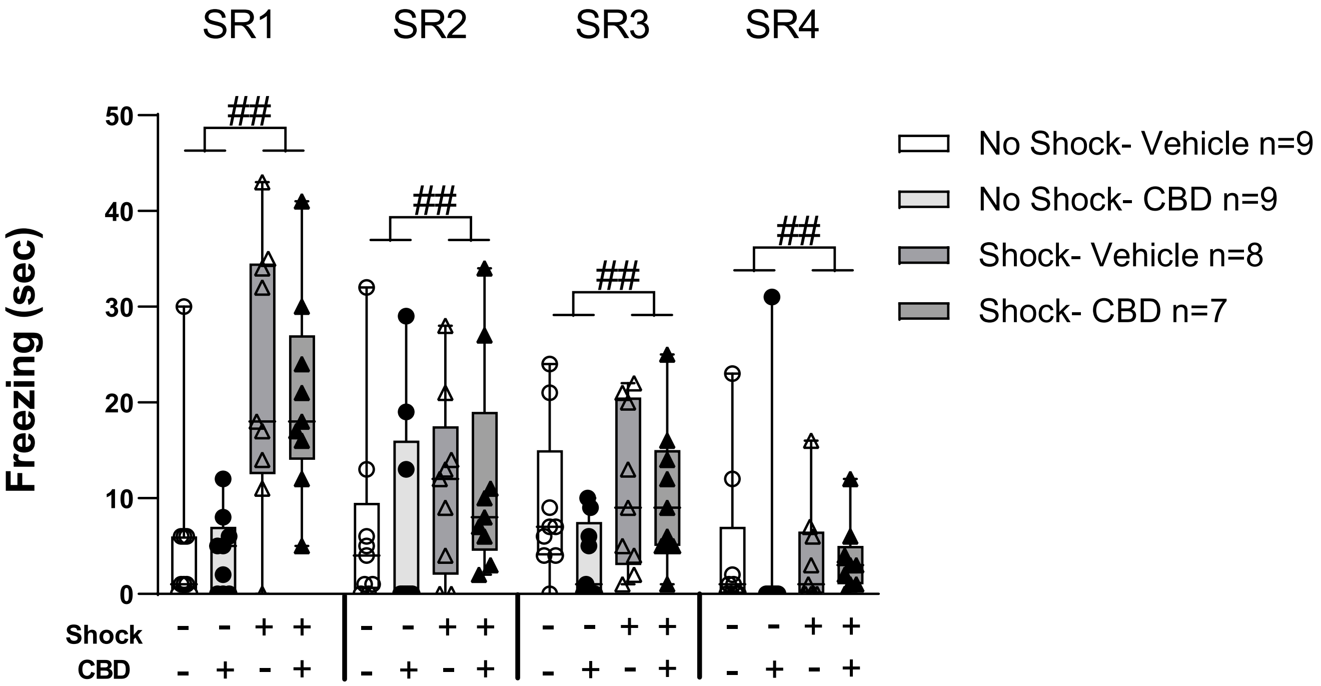

Figure S2. Freezing behavior during the situational reminders (SRs). Rats were exposed to SRs four times over a 28-day period, specifically on days 7, 14, 21, and 28. During each SR, the rats spent one minute in the light start chamber with the gate closed, preventing entry into the shock compartment and thus avoiding extinction. The shocked groups exhibited increased freezing behavior compared to the no-shock groups across all SRs. ##, $p < 0.01$ indicates statistical significance in the main effects.

Table S1. Distribution of estrus phases in female rats on shock day and the first day of extinction. The table shows an even distribution of female rats across the diestrus, proestrus, estrus, and metestrus phases within each group.

	Estrus phase	Shock day	Ext 1
No Shock-Vehicle	Diestrus	0	3
	Proestrus	2	3
	Estrus	2	1
	Metestrus	5	2
No Shock-CBD	Diestrus	0	0
	Proestrus	5	4
	Estrus	2	5
	Metestrus	2	0
Shock-Vehicle	Diestrus	0	0
	Proestrus	3	1
	Estrus	4	5
	Metestrus	2	3
Shock-CBD	Diestrus	2	0
	Proestrus	0	3
	Estrus	2	2
	Metestrus	5	4

CBD: cannabidiol; Ext: extinction day.

Table S2. Pearson bivariate correlation between estrus phase on the first day of extinction and the latency to enter the dark chamber on Ext 1 in shock- and situational reminders-exposed female rats.

	**Latency to enter the dark chamber on Ext 1**
**Estrus phase**	r = 0.319
	p = 0.066

Ext: extinction day.
